# Comparative analysis based on replication banding reveals the mechanism responsible for the difference in the karyotype constitution of treefrogs *Ololygon* and *Scinax* (Arboranae, Hylidae, Scinaxinae)

**DOI:** 10.3897/CompCytogen.11(2).11254

**Published:** 2017-04-18

**Authors:** Simone Lilian Gruber, Gabriela Isabela Gomes de Oliveira, Ana Paula Zampieri Silva, Hideki Narimatsu, Célio Fernando Baptista Haddad, Sanae Kasahara

**Affiliations:** 1 UNESP, Universidade Estadual Paulista, Instituto de Biociências, Departamento de Biologia, Av. 24A, 1515, 13506-900, Rio Claro, SP, Brazil; 2 UNESP, Universidade Estadual Paulista, Instituto de Biociências, Departamento de Zoologia, Av. 24A, 1515, 13506-900, Rio Claro, SP, Brazil

**Keywords:** Anura, Hylidae, 5-bromodeoxiuridine, comparative cytogenetics, NOR, karyotype evolution

## Abstract

According to the recent taxonomic and phylogenetic revision of the family Hylidae, species of the former *Scinax catharinae* (Boulenger, 1888) clade were included in the resurrected genus *Ololygon* Fitzinger, 1843, while species of the *Scinax ruber* (Laurenti, 1768) clade were mostly included in the genus *Scinax* Wagler, 1830, and two were allocated to the newly created genus *Julianus* Duellman et al., 2016. Although all the species of the former *Scinax* genus shared a diploid number of 2n = 24 and the same fundamental number of chromosome arms of FN = 48, two karyotypic constitutions were unequivocally recognized, related mainly to the distinct size and morphology of the first two chromosome pairs. Some possible mechanisms for these differences had been suggested, but without any experimental evidence. In this paper, a comparison was carried out based on replication chromosome banding, obtained after DNA incorporation of 5-bromodeoxiuridine in chromosomes of *Ololygon* and *Scinax*. The obtained results revealed that the loss of repetitive segments in chromosome pairs 1 and 2 was the mechanism responsible for karyotype difference. The distinct localization of the nucleolus organizer regions in the species of both genera also differentiates the two karyotypic constitutions.

## Introduction

The family Hylidae was recently revised by Duellman et al. (2016), who proposed a new phylogenetic tree based on 503 species and 16, 128 aligned sites of 19 genes. According to this phylogeny, the families Hylidae, Pelodryadidae, and Phyllomedusidae were placed in an unranked taxon named Arboranae, a new subfamily Scinaxcinae and a new genus *Julianus* Duellman, Marion, Hedges, 2016 were created, and the genus *Ololygon* Fitzinger, 1843 was resurrected, among other modifications. The hylids *Scinax* Wagler, 1830 and *Ololygon* are included in Scinaxinae and form a cluster along with *Julianus* and *Sphaenorhynchus* Tschudi, 1838. The support value for the cluster of the *Julianus*, *Ololygon*, and *Scinax* species was high (97%), but the placement of *Sphaenorhynchus* was low (49%), thus, according to Duellman et al. (2016), this relationship is still uncertain and needs to be reviewed.

Over the years, the hylid frogs of the former *Scinax* genus have been subjected to several taxonomic and phylogenetic reviews (Faivovich 2002, Faivovich et al. 2005, Wiens et al. 2010), with a large sample of specimens based mainly on molecular sequencing of mitochondrial and nuclear genes. In the classification proposed by Duellman et al. (2016), the newly constituted genus *Scinax* includes the species of the *Scinax ruber* (Laurenti, 1768) clade, with the exception of *S. uruguayus* (Schmidt, 1944) and *S. pinima* (Bokermann & Sazima, 1973) (from the former *S. uruguayus* group), both assigned to the genus *Julianus*, along with *Sphaenorhynchus*, whereas *Ololygon* comprises species of the former *Scinax catharinae* (Boulenger, 1888) clade.

The *Ololygon* and *Scinax* species are distributed across the Americas from Mexico to Argentina and Uruguay, and the islands of Tobago, Trinidad, and Saint Lucia (Duellman et al. 2016, Frost 2016). They are abundant in Brazil, with great diversity and endemism in the Atlantic Forest and Cerrado domains (Araújo-Vieira et al. 2016, Lourenço et al. 2016). They are considered complex groups due to great morphological similarity between individuals, which hinders proper species identification and determination of clear synapomorphies for taxonomic description (Pombal Jr. et al. 1995, Lourenço et al. 2016).

Early researches on the cytogenetics of the former *Scinax* species determined the chromosome number by observing mitotic or meiotic cells. All of the species exhibited a diploid number of 2n = 24 bi-armed chromosomes, corresponding to the fundamental number of FN = 48 chromosome arms (revisions in Catroli and Kasahara 2009 and in Peixoto et al. 2015). The work of Cardozo et al. (2011) stands out for its inclusion of chromosomal data obtained from a large sample of 28 representatives, and because the results were analyzed for the first time from an evolutionary point of view provided by available phylogenies. This work highlighted a karyotype differentiation between the *S. catharinae* and the *S. ruber* clades, regarding the morphology and size of the first two chromosome pairs, the position of Ag-NORs, and the amount of C-banded heterochromatin. Additionally, the combined analysis with phylogeny allowed these authors to establish that the characteristic Ag-NORs marker in small pair 11, as exhibited by the species in the *S. ruber* clade, was considered a plesiomorphy, whereas the location in pair 6 in the species of the *S. catharinae* clade is a derivative condition. Only recently, a small number of species of *Scinax* (*lato sensu*) have had their karyotypic analyses extended through the use of more advanced cytogenetic techniques, such as investigation of the nature of repetitive regions using fluorochrome staining or fluorescent *in situ* hybridization (FISH) with microsatellite probes, and the location of ribosomal or telomeric sequences using the FISH technique (Oliveira 2011, Nogueira et al. 2015a, Nogueira et al. 2015b, Peixoto et al. 2015, Peixoto et al. 2016).

In the present work our efforts were the use of replication banding after 5-bromodeoxiuridine treatment, an useful approach to identify homeologous chromosomes (Kasahara et al. 2003, Silva et al. 2004, Gruber et al. 2007, Gazoni et al. 2012, Gruber et al. 2012, Gruber et al. 2013). This methodology is very effective for comparative analyses in fishes, amphibians, and reptiles due to the difficulty in producing classical Q, G, and R banding patterns in the chromosomes of the ectothermic vertebrates (Sumner 2003).

Replication banding was fundamental to provide evidence of the structural rearrangement responsible for the difference in the karyotypes between *Ololygon* and *Scinax* species, thereby contributing for clarify the phylogenetic relationships between these two genera.

## Material and methods

Five species currently included in the genus *Ololygon*, *O. albicans* (Bokermann, 1967), *O. argyreornata* (Miranda-Ribeiro, 1926), *O. hiemalis* (Haddad & Pombal, 1987), *O. littoralis* (Pombal & Gordo, 1991), and *O. obtriangulata* (Lutz, 1973), and six species of genus *Scinax*, *S. caldarum* (Lutz, 1968), *S. crospedospilus* (Lutz, 1925), *S. eurydice* (Bokermann, 1968), *S. fuscovarius* (Lutz, 1925), *S. hayii* (Barbour, 1909), and *S. similis* (Cochran, 1952) were collected from Brazilian locations (Table 1) with the consent and approval of Instituto Chico Mendes de Conservação da Biodiversidade (38827-2). The classification adopted in the present work is based on the Amphibian Species of the World electronic database (Frost 2016).

**Table 1.** Analyzed species, voucher number (CFBH), sample size, sex, and collection locality.

Species	Voucher Number (CFBH)	Sample size	Sex	Collection locality
*Ololygon albicans*	10178^3^	1	Male	Petrópolis, RJ 22°30'17"S; 43°10'56"W
*Ololygon argyreornata*	17289^3^, 17290^3^	2	Males	Ilha do Cardoso, SP 25°07'31"S; 47°58'06"W
*Ololygon hiemalis*	28591^2^, 36231^2^, 36233^2^	3	Males	Mogi Guaçu, SP 22°21'37"S; 57°07'07"W
*Ololygon littoralis*	40631^2^	1	Male	Bertioga, SP 23°56'27"S; 45°19'48"W
	22378^3^	1	Male	Ubatuba, SP 23°26'19"S; 45°05'25"W
*Ololygon obtriangulata*	CFBHT^*^20329^2^	1	Female	Biritiba Mirim, SP 23°34'17"S; 46°02'15"W
*Scinax caldarum*	22552^1^	1	Male	Poços de Caldas, MG 21°47'18"S; 46°33'45"W
*Scinax crospedospilus*	36201^1^, 36202^1^	2	Males	Mogi das Cruzes, SP 23°31'29"S; 46°11'14"W
*Scinax eurydice*	16736^3^	1	Male	Serra do Japi, Jundiaí, SP 37°25'818"S; 122°05'36"W
*Scinax fuscovarius*	22415^2^	1	Female	Biritiba Mirim, SP 23°34'17"S; 46°02'15"W
	28730^4^	1	Male	Socorro, SP 22°35'29"S; 46°31'44"O
*Scinax hayii*	24216^2^	1	Male	Biritiba Mirim, SP 23°34'17"S; 46°02'15"W
	28588^2^	1	Male	Mogi das Cruzes, SP 23°31'29"S; 46°11'14"W
*Scinax similis*	5933^3^, 5932^3^	2	Females	Três Marias, MG 18°12'43.3"S; 45°13'19"W

^1^-species with first karyotype description; ^2^-specimens belonging to species already described in Cardozo et al. (2011) but collected in non studied populations; ^3^-additional banding data of specimens from the analyzed sample in Cardozo et al. (2011); ^4^-specimen reanalyzed from Kasahara et al. (2003) sample;^*^ identification based on the COI sequencing from tissue sample.

The sample includes species whose chromosomes are described for the first time in this paper: *Scinax caldarum* and *S. crospedospilus*; specimens belonging to species already described in Cardozo et al. (2011) but collected in non studied populations: *Ololygon hiemalis*, *O. littoralis*, *O. obtriangulata*, *S. fuscovarius*, and *S. hayii*; additional banding data of specimens from the analyzed sample in Cardozo et al. (2011): *O. albicans*, *O. argyreornata*, *O. littoralis*, *S. eurydice*, and *S. similis*; and specimen CFBH 28730 of *S. fuscovarius*, the cytogenetic data of which were previously described in Kasahara et al. (2003): the replication bands of this specimen were reanalyzed herein because its high resolution banding was more appropriate for comparative analysis.

The animals were identified by Dr. Célio F. B. Haddad and the vouchers were fixed in formalin (10%), preserved in 70% ethanol and deposited in the amphibian collection CFBH of the Departamento de Zoologia, Instituto de Biociências, UNESP, Rio Claro, SP, Brazil. Identification of the specimen CFBHT 20329 was confirmed by COI sequencing from muscle sample collected soon after the animal euthanasia and preserved in 70% ethanol. Direct cytological preparations were obtained from bone marrow, liver, and also the testes of male samples (Baldissera Jr et al. 1993). Spleen was eventually used depending on its size and also dissociated in hypotonic solution. For some animals, cytological preparations were made from the intestinal epithelium (Schmid 1978) with modifications. For direct preparation, the animals received a previous injection of 0.1% colchicine during 2 to 6 hours to improve the quality and number of metaphases. Some specimens were submitted to *in vivo* treatment for 13 to 21 hours after injection of 5-bromodeoxiuridine (BrdU) plus 5-fluorodeoxyuridine (FudR) (10mg BrdU and 0.5mg FudR in 2mL 0.9% NaCl solution), in the proportion of 0.1mL/10g body weight (Silva et al. 2000). Briefly, the steps in this process are incorporation of BrdU during DNA replication, after *in vivo* injection or *in vitro* treatment during lymphocytes or fibroblast cultures, followed by differentiation of replication banding patterns with Hoechst 33258 staining, black light irradiation, and Giemsa staining (Dutrillaux and Couturier 1981, Sumner 2003).

Mitotic and meiotic preparations were analyzed after Giemsa staining. Mitotic chromosomes were also submitted to the techniques of silver impregnation (Ag-NOR) (Howell and Black 1980), C-banding (Sumner 1972), and fluorochrome staining with chromomycin A_3_ (CMA_3_) and 4’,6-diamidino-2-phenylindole (DAPI) (Christian et al. 1998). The differentiation of replication bands after BrdU incorporation was achieved using Fluorochrome Plus Giemsa (FPG) staining as described by Dutrillaux and Couturier (1981) and modified by Kasahara et al. (1998). FISH was performed with an HM123 ribosomal probe (Meunier-Rotival et al. 1979, Pinkel et al. 1986), and a telomeric probe (DAKO Cytomation Denmark A/S), following manufacturer’s instructions.

Chromosomes were analyzed under standard and UV light. The best mitotic and meiotic cells were photographed using an Olympus BX51 microscope and digital capture system DP71. Copies of the material were obtained digitally. Karyograms were assembled according to the morphology of the chromosomes in decreasing order of size (Table 2). The bi-armed chromosomes were classified as metacentric, submetacentric or subtelocentric (Green and Sessions 1991, 2007).

**Table 2.** Relative length (RL), centromeric index (CI), and nomenclature for centromeric position (CP) on mitotic chromosomes of *Ololygon* and *Scinax*, according to Green and Sessions (1991, 2007).

Species		Chromosome pair											
		1	2	3	4	5	6	7	8	9	10	11	12
*O. albicans*	RL	12.54	12.10	10.52	10.37	9.22	8.93	8.93	6.20	6.05	5.48	4.76	4.90
	CI	0.349	0.300	0.314	0.222	0.235	0.250	0.250	0.455	0.382	0.500	0.500	0.500
	CP	**sm**	**sm**	**sm**	**st**	**sm**	**st**	**sm**	**m**	**m**	**m**	**m**	**m**
*O. argyreornata*	RL	11.65	10.67	11.59	10.45	9.53	8.87	8.17	7.35	6.37	6.31	4.84	4.19
	CI	0.363	0.340	0.274	0.348	0.311	0.265	0.250	0.429	0.500	0.500	0.444	0.429
	CP	**sm**	**sm**	**sm**	**sm**	**sm**	**sm**	**sm**	**m**	**m**	**m**	**m**	**m**
*O. hiemalis*	RL	13.33	11.16	10.23	9.92	8.99	9.61	8.84	6.20	6.20	5.89	4.96	4.65
	CI	0.300	0.333	0.294	0.290	0.286	0.290	0.310	0.500	0.400	0.500	0.500	0.500
	CP	**sm**	**sm**	**sm**	**sm**	**sm**	**sm**	**sm**	**m**	**m**	**m**	**m**	**m**
*O. littoralis*	RL	12.30	11.50	10.70	10.70	9.36	8.02	8.56	7.09	6.15	5.75	5.35	4.55
	CI	0.292	0.364	0.282	0.250	0.333	0.310	0.313	0.462	0.455	0.435	0.500	0.500
	CP	**sm**	**sm**	**sm**	**sm**	**sm**	**sm**	**sm**	**m**	**m**	**m**	**m**	**m**
*O. obtriangulata*	RL	11.71	11.31	10.50	9.96	9.96	9.42	7.81	6.73	6.19	5.92	5.25	5.25
	CI	0.349	0.333	0.300	0.211	0.263	0.257	0.267	0.500	0.417	0.455	0.500	0.474
	CP	**sm**	**sm**	**sm**	**st**	**sm**	**sm**	**sm**	**m**	**m**	**m**	**m**	**m**
*S. caldarum*	RL	13.87	12.14	10.40	9.25	9.25	8.67	8.09	7.80	6.07	5.20	4.62	4.62
	CI	05.00	0.455	0.444	0.250	0.375	0.333	0.429	0.467	0.400	0.500	0.500	0.375
	CP	**m**	**m**	**M**	**sm**	**sm**	**sm**	**m**	**m**	**m**	**m**	**m**	**m**
*S. crospedospilus*	RL	14.53	12.50	10.47	10.81	8.28	8.28	6.59	6.59	5.91	5.74	5.24	5.07
	CI	0.476	0.444	0.344	0.281	0.320	0.333	0.400	0.474	0.474	0.471	0.500	0.500
	CP	**m**	**m**	**sm**	**sm**	**sm**	**sm**	**m**	**m**	**m**	**m**	**m**	**m**
*S. eurydice*	RL	15.51	11.57	10.16	10.01	9.31	8.60	7.05	6.35	5.50	5.64	5.22	5.08
	CI	0.500	0.455	0.333	0.316	0.250	0.333	0.407	0.478	0.500	0.450	0.444	0.444
	CP	**m**	**m**	**sm**	**sm**	**sm**	**sm**	**m**	**m**	**m**	**m**	**m**	**m**
*S. fuscovarius*	RL	16.05	12.23	10.39	10.39	8.56	7.73	6.70	6.11	5.81	5.50	5.47	5.04
	CI	0.481	0.429	0.353	0.294	0.286	0.308	0.385	0.500	0.444	0.500	0.444	0.471
	CP	**m**	**m**	**sm**	**sm**	**sm**	**sm**	**sm**	**m**	**m**	**m**	**m**	**m**
*S. hayii*	RL	13.73	11.16	10.94	10.30	9.23	8.15	7.94	7.08	6.22	5.36	5.36	4.51
	CI	0.500	0.462	0.370	0.417	0.381	0.500	0.444	0.500	0.429	0.500	0.462	0.400
	CP	**m**	**m**	**sm**	**m**	**sm**	**m**	**m**	**m**	**m**	**m**	**m**	**m**
*S. similis*	RL	15,20	12.94	12.01	10.39	7.45	7.89	7.06	6.13	5.98	5.59	4.95	4.41
	CI	0.500	0.419	0.458	0.227	0.301	0.354	0.400	0.462	0.385	0.483	0.404	0.500
	CP	**m**	**m**	**M**	**st**	**sm**	**sm**	**m**	**m**	**sm**	**m**	**m**	**m**

m = metacentric; sm = submetacentric; st = subtelocentric.

## Results

**Figure 1.**
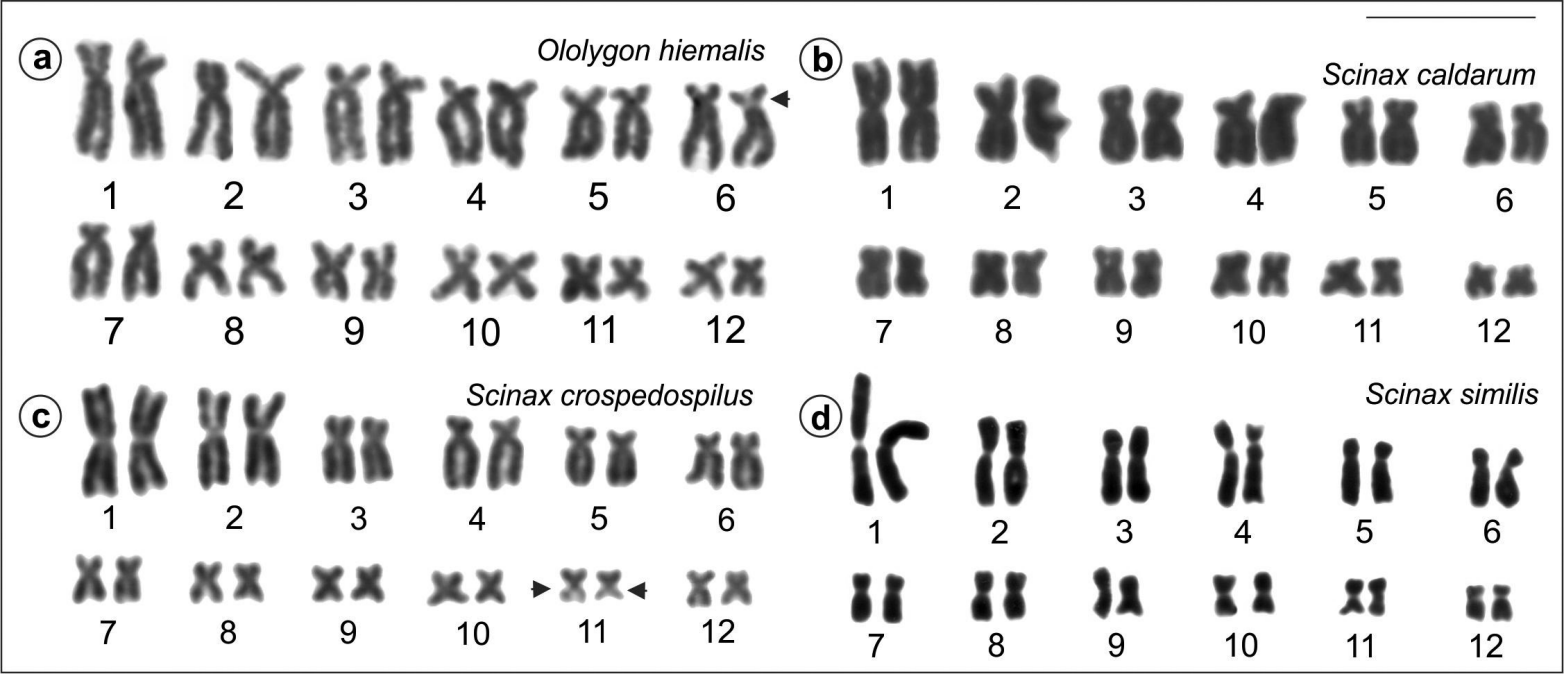
Giemsa stained karyotypes of *Ololygon* and *Scinax* species. **a** *O. hiemalis*, male **b** *S. caldarum*, male **c** *S. crospedospilus*, male **d** *S. similis*, female, with heteromorphic chromosomes pairs 3 and 4. Secondary constrictions (arrowhead) are visible on chromosome 6 in **a** and on chromosomes 11 in **c**. Bar = 10 μm.

All investigated specimens presented 24 bi-armed chromosomes. In *Ololygon* species, chromosome pairs 1 and 2 were submetacentric with a slight size difference between them, while in the species of *Scinax* genus chromosome pairs 1 and 2 were metacentric with great size difference. For illustration, the Giemsa-stained karyotypes of one of the species of *Ololygon* (*O. hiemalis*) from a population not studied before and of the two species of *Scinax* (*S. caldarum* and *S. crospedospilus*) karyotyped for the first time are presented in Figure 1. The karyotype of one of the specimens of *S. similis* with heteromorphic chromosome pairs 3 and 4 is also shown. The remaining species had the standard stained karyotypes published previously in Cardozo et al. (2011).

**Figure 2.**
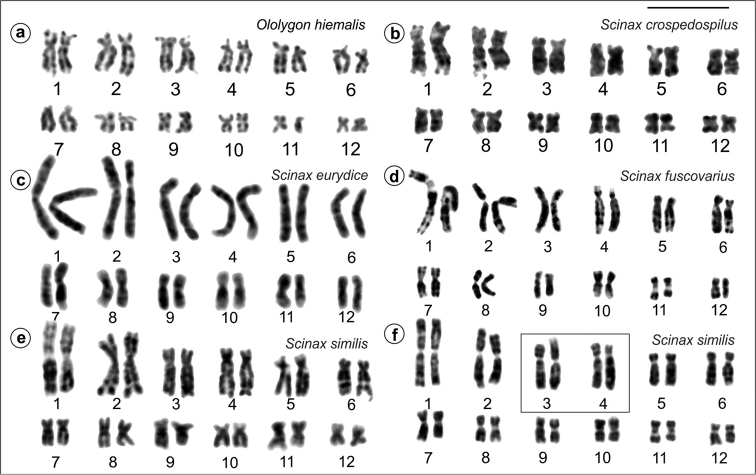
Replication banding in *Ololygon* and *Scinax* species. **a** *O. hiemalis* **b** *S. crospedospilus* **c** *S. eurydice* **d** *S. fuscovarius* **e–f** *S. similis*. Note heteromorphic chromosome pairs 3 and 4 in inset. Bar = 10 μm.

**Figure 3.**
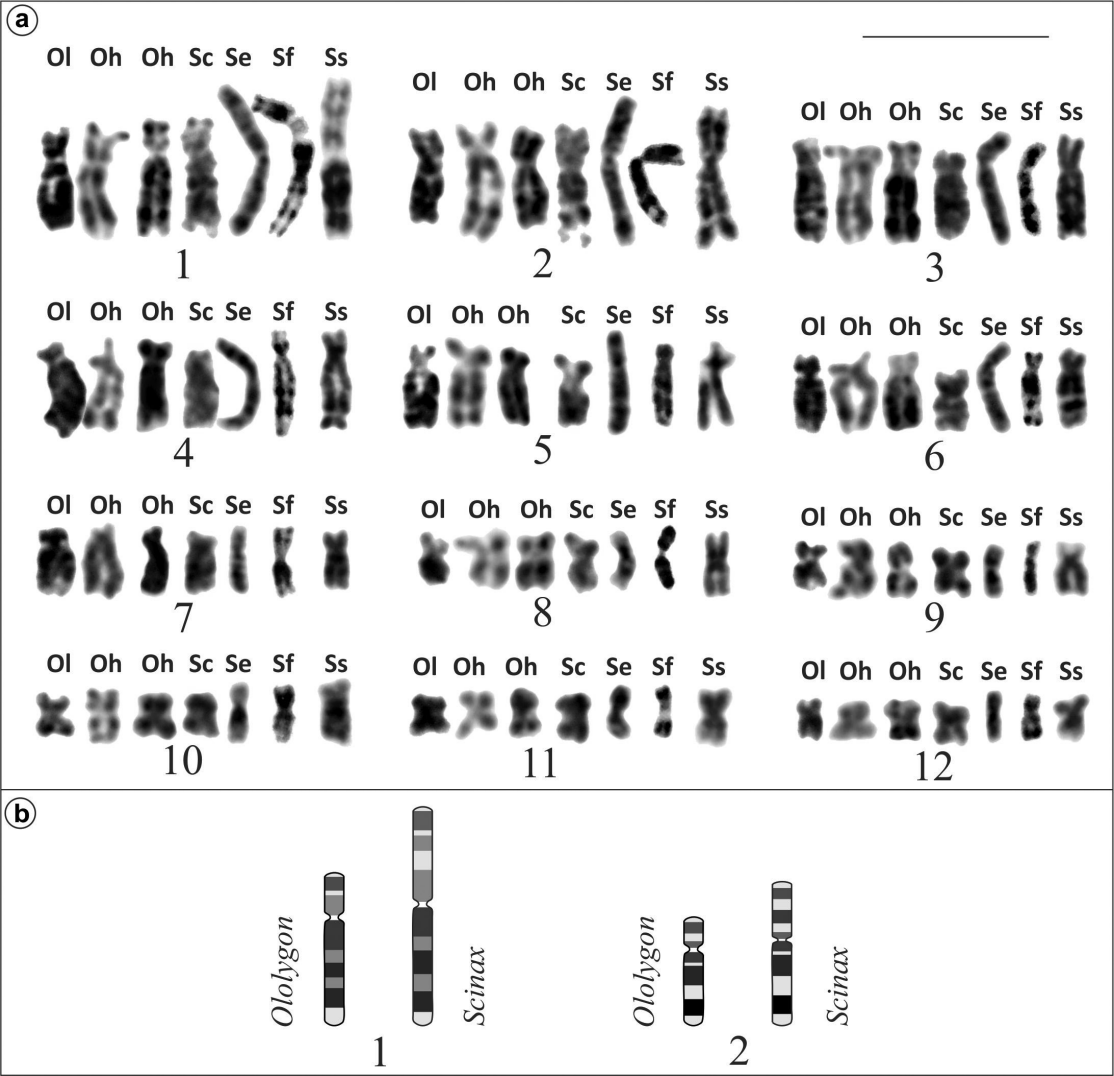
**a** Comparison of replication bands of the chromosomes of: **Ol** = *O. littoralis*; **Oh** = *O. hiemalis* (two distinct metaphases of the same specimen); **Sc** = *S. crospedospilus*; **Se** = *S. eurydice*; **Sf** = *S. fuscovarius*; **Ss** = *S. similis*. **b** Ideogram of chromosomes 1 and 2 of *Ololygon* and *Scinax* evidencing loss of segment in the short arms of both chromosomes in *Ololygon*. Bar = 10 μm.

Replication banding patterns for the chromosomes of *O. hiemalis*, *S. crospedospilus*, *S. eurydice*, *S. fuscovarius*, and for two specimens of *S. similis* are presented in Figure 2. Comparison of the banded chromosomes of species of *Ololygon* and *Scinax* genera revealed close correspondence for the majority of their chromosome pairs, except for the chromosomes of pairs 1 and 2 (Figure 3a, b). In species of *Ololygon*, the short arms of chromosomes 1 and 2 are respectively smaller than the short arms of chromosomes 1 and 2 of *Scinax* species, whereas the long arms of both chromosomes have the same size and corresponding bands.

**Figure 4.**
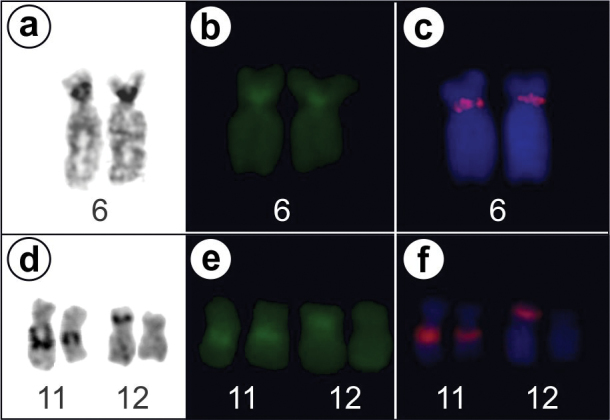
Nucleolus organizer regions in *Ololygon obtriangulata* (**a, b, c**) and *Scinax fuscovarius* (**d, e, f**) identified by **a, d** silver impregnation **b, e** CMA_3_ staining, and **c, f** FISH with rDNA probe.

Nucleolus organizer regions were located on pair 6 for the *Ololygon* species and in pair 11 for the *Scinax* species. Figure 4 shows Ag-NOR, CMA_3_ staining, and FISH using an rDNA probe in *O. obtriangulata* and *S. fuscovarius* CFBH 22415. In the CFBH 22415 specimen an additional Ag-NOR site was observed in one of the chromosomes 12, and this was confirmed after both CMA_3_ fluorochrome staining and FISH labeling.

**Figure 5.**
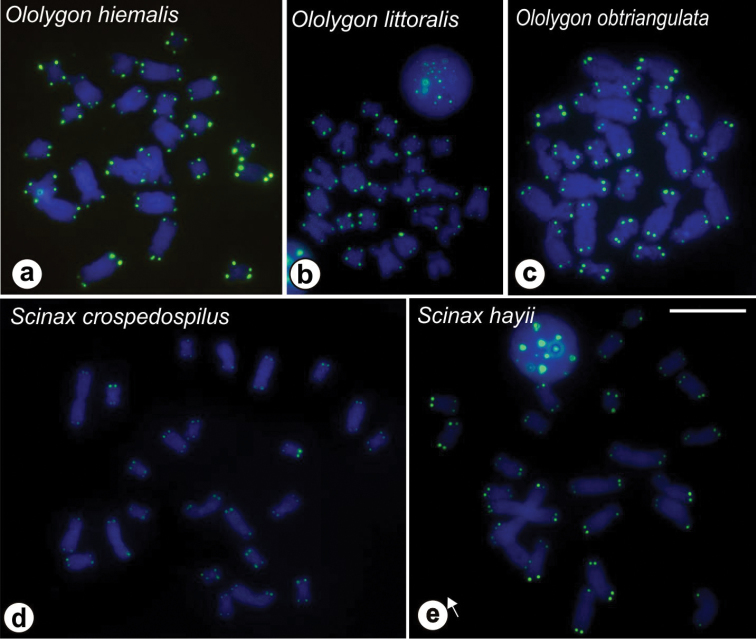
FISH with telomeric probes in *Ololygon* and *Scinax* species. **a** *O. hiemalis* **b** *O. littoralis* **c** *O. obtriangulata* **d** *S. crospedospilus* **e** *S. hayii*. Bar = 10 μm.

**Figure 6.**
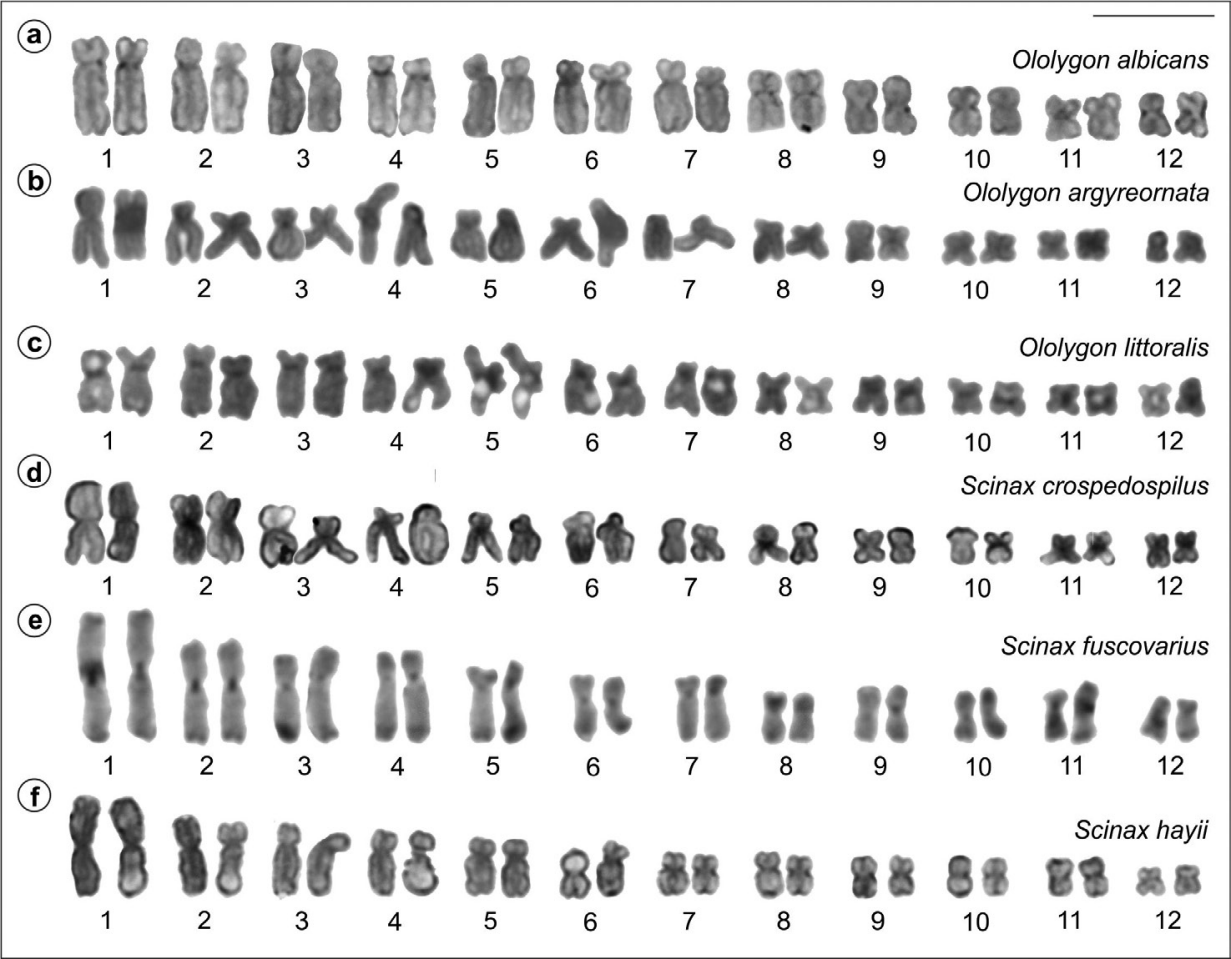
C-banding of *Ololygon* and *Scinax* species. **a** *O. albicans* **b** *O. argyreornata* **c** *O. littoralis* **d** *S. crospedospilus* **e** *S. fuscovarius* **f** *S. hayii*. Bar = 10 μm.

**Figure 7.**
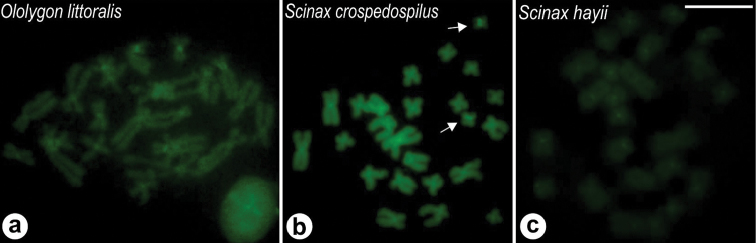
Cromomycin A_3_ staining in *Ololygon* and *Scinax* species. **a** *O. littoralis* **b** *S. crospedospilus* **c** *S. hayii*. NOR-bearing chromosomes in **b** (arrow). Bar = 10 μm.

Chromosome preparations of *O. hiemalis*, *O. littoralis*, *O. obtriangulata*, *S. crospedospilus*, and *S. hayii* were submitted to hybridization with a telomeric probe and only the terminal regions of all chromosomes in these species were labeled (Figure 5). No probe hybridization was observed outside the telomeres in any of these species. The C-banding technique conducted on the chromosome preparations of *O. albicans*, *O. argyreornata*, *O. littoralis*, *S. crospedospilus*, *S. hayii*, and *S. fuscovarius* revealed poorly defined C-bands, which were faintly stained, mainly in the centromeric region of some chromosomes of the species (Figure 6). Fluorochrome staining using CMA_3_ showed bright labeling of the NORs in the species of the sample and in some of them, such as *O. littoralis*, *S. crospedospilus*, and *S. hayii*, the centromeric regions also appeared less brightly stained (Figure 7). DAPI fluorochrome staining revealed that the chromosomes of the species presented a homogeneously stained appearance, with no particularly fluorescent region.

**Figure 8.**
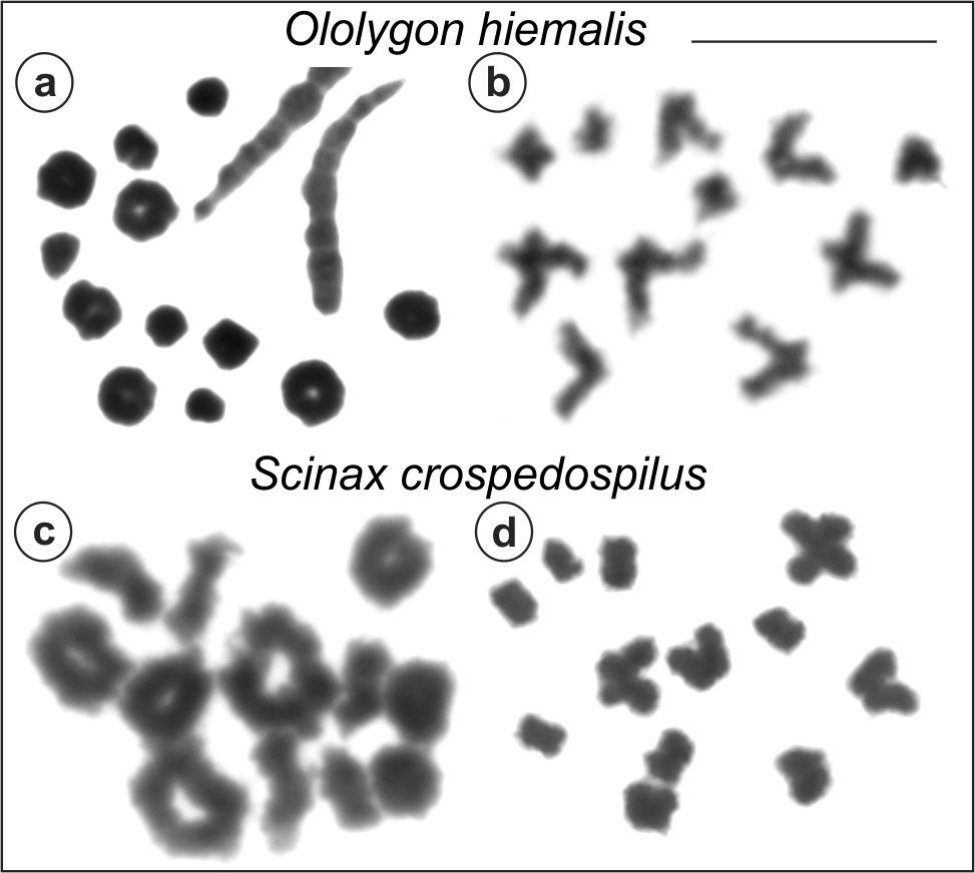
Meiotic cells in males of *Ololygon hiemalis* **(a, b)** and *Scinax crospedospilus* **(c, d)**. **a, c** Metaphase I with 12 bivalents **b, d** Metaphase II with 12 chromosomes. Bar = 10 μm.

Testis preparations were obtained from male specimens of *O. argyreornata*, *O. hiemalis*, *O. littoralis*, *S. caldarum*, *S. crospedospilus*, *S. eurydice*, and *S. hayii*. In all of them, metaphase I cells presented 12 bivalents, whereas metaphase II cells presented 12 chromosomes, as shown in Figure 8 for one species of each analyzed genus, namely *O. hiemalis* and *S. crospedospilus*. No heteromorphic bivalent that could suggest the presence of differentiated sex chromosomes of the XY type was observed in any of the analyzed species.

## Discussion

Although *Ololygon* and *Scinax* species share the same diploid and fundamental numbers, two karyotypic constitutions were unequivocally distinguished by visual inspection of standard stained chromosomes as well as comparative measurements. One of these karyotypes is characteristic for all five species, currently recognized as belonging to the genus *Ololygon* (Duellman et al. 2016, Frost 2016), but formerly assigned to the *Scinax catharinae* clade. The other karyotypic constitution is exhibited by the six remaining species of the sample, currently recognized as belonging to the genus *Scinax* but previously included in the *Scinax ruber* clade.

The difference between the two karyotypes, which is related to the morphology and size of chromosome pairs 1 and 2, had already been indicated in one of the first cytogenetic studies performed in the 1970’s by Bogart (1973) when the species of both taxa were assigned to the genus *Hyla*. Nevertheless, a possible third karyotypic constitution that had been suggested by that author was not confirmed here or by Cardozo et al. (2011) who studied a large sample of species of *Scinax* (*lato sensu*). Subsequent studies (Oliveira 2011, Nogueira et al. 2015a, Nogueira et al. 2015b, Peixoto et al. 2015, Lourenço et al. 2016, and Peixoto et al. 2016) confirmed that the two chromosome constitutions remained invariably the same, indicating that the karyotypes are relatively conserved within each of the two taxonomic groups.

Although without experimental evidence, Cardozo et al. (2011) suggested possible mechanisms responsible for the morphological and size differences of pairs 1 and 2 in the two karyotypic constitutions, such as pericentric inversion, addition/loss of repetitive sequences or unidentified structural chromosome alterations. Reproducible banding patterns obtained by BrdU incorporation in the present sample were sufficient for precise pairing of the homologous chromosomes. It was also useful in interspecific comparative analysis for identifying homeologous sets of chromosomes, mainly those which were large or medium sized. Pericentric inversion could be ruled out, but not the addition or loss of repetitive chromosome segments, due to the different size of the short arms of chromosomes 1 and 2 in the two compared karyotypic constitutions. This is quite evident in the case of chromosome 1 and less noticeable in the case of chromosome 2. Nevertheless, it is important to remark that although the poor quality of our results these repetitive regions were not positively stained after C-banding data, a finding also described by Cardozo et al. (2011) analyses.

The probable direction of chromosome evolution, in other words, the loss or gain of repetitive segments in chromosome pairs 1 and 2, is suggested in light of the cytogenetic data of *Ololygon* and *Scinax* species, and the phylogeny of Faivovich et al. (2005). Similar integrative taxonomic approach was carried out by Lourenço et al. (2015) for amphibians and Mezzasalma et al. (2015) for reptiles. As already pointed out by Cardozo et al. (2011) and subsequently confirmed in other works, the species of *Ololygon* (the former *Scinax catharinae* clade) have NORs in chromosome pair 6, whereas the species of *Scinax* (the former *Scinax ruber* clade) generally have this marker in the chromosome pair 11, which also indicates the conservation of this character to cytogenetically differentiate species of both groups. It is important to note that a chromosome constitution with 2n = 24, including the position of NORs, which is characteristic of currently recognized *Scinax* species, is observed in the vast majority of representatives of hylinaes sharing a 2n = 24 (Kasahara et al. 2003, Cardozo et al. 2011). It can consequently be assumed that the chromosome constitution characteristic of the *Scinax* genus (the former *Scinax ruber* clade) would be the ancestor, while that presented by the species of *Ololygon* (the former *Scinax catharinae* clade) would be the derived condition, as it was previously pointed out by Cardozo et al. (2011) In this case, the process of differentiating the karyotype of both groups was probably the deletion and not the addition of repetitive DNA in the short arms of chromosomes 1 and 2, in an ancestral karyotypic form similar to that exhibited by the current *Scinax* species and other Hylinae. Not only do the present data provided the probable direction of the chromosome evolution, they also support the subdivision of the former *Scinax* into two genera, as previously suggested by Pombal Jr et al. (1995), Faivovich (2002), based on morphology, bioacoustics, among others characters and effectively established by Duellman et al. (2016). In this case, the most derived group is actually represented by the species of *Ololygon*.

Obtaining replication bands along chromosomes was helpful to clarify the heteromorphism in pairs 3 and 4 in one of the two specimens of the sample of *Scinax similis* described in Cardozo et al. (2011). Although the Giemsa-stained karyotypes strongly suggested that this heteromorphism resulted from reciprocal translocation, in our study it was definitely confirmed by comparative replication banding analysis. The structural rearrangement is most likely a sporadic finding that can occur in natural populations (Siqueira et al. 2004, Schmid et al. 2010). The results obtained so far using silver impregnation revealed that this technique is widely useful for characterizing the species of *Ololygon* and *Scinax* (the former *S. catharinae* and *S. ruber* clades, respectively). In a few cases, there were exceptions to the localization of Ag-NORs, which were found to be discrepancies in ordering the chromosomes in the karyograms or due to true changes in the position of this chromosome marker which can eventually occur in natural populations (Cardozo et al. 2011). Another finding reported by these authors refers to the occurrence of multiple Ag-NORs in some species of the sample. In the specimen CBFH 22415 of *S. fuscovarius* herein analyzed three Ag-NORs per metaphase (two in chromosome pair 11 and one in a single homologue of pair 12) were observed but FISH using an rDNA probe confirmed the additional labeling in chromosome 12 as true NOR. This variant pattern of NOR may be a sporadic derived condition possibly characteristic for the population ascertained for the first time in the present work.

Based on C-banding data of species of the former *Scinax* clades, Cardozo et al. (2011) suggested that the amount of centromeric C-banded heterochromatin would also represent a difference between the karyotypes of the analyzed species. Nevertheless, the data obtained herein for species of *Ololygon* and *Scinax* and those of Nogueira et al. (2015a), Nogueira et al. (2015b), and Peixoto et al. (2015) for the species of the former *Scinax catharinae* and *Scinax ruber* clades are inconsistent for a clear-cut differentiation of the two karyotypic constitutions with this chromosome marker.

The FISH technique using telomeric probes tested in the chromosome preparations of *Ololygon* and *Scinax* of our sample, which could potentially clarify the occurrence of structural rearrangement during the chromosome evolution, as demonstrated by Fagundes and Yonenaga-Yassuda (1998) in Akodont rodents, did not provide any evidence.

## Conclusions

Our cytogenetic data confirm the loss of repetitive sequence in the short arms of chromosomes 1 and 2 as the mechanism responsible for the difference in the karyotypic constitutions of *Ololygon* and *Scinax*. Nevertheless, relationships in Scinaxcinae species are not easy to interpret. Despite the taxonomic difficulties, increasing the number of analyzed species and using more cytological markers based on molecular cytogenetics methodologies will eventually help to clarify phylogenetic questions within *Ololygon*, *Scinax*, and related genera.
